# Management of Mandibular Compound Odontoma With Numbness in the Lower Jaw

**DOI:** 10.7759/cureus.51315

**Published:** 2023-12-30

**Authors:** Khalid A Binzamil, Ahmed S Almslam, Abdullah A Baaboud, Abdulaziz A Altwirki, Atif A Alghamdi, Ahmad Al-Omar, Reem S Almslam

**Affiliations:** 1 Dentistry, King Saud University, Riyadh, SAU; 2 Surgery, King Saud University, Riyadh, SAU

**Keywords:** impacted canine, retained deciduous teeth, numbness in lower jaw, odontomas, odontogenic tumor, compound odontoma

## Abstract

Odontomas are considered to be among the more common odontogenic tumors in the oral cavity. Several authors classify them as hamartomas instead of actual tumors. Odontomes' precise etiology is still unknown. The majority of odontomas are found during routine radiography studies and are asymptomatic. Odontomes typically cause disruptions to the teeth's eruption, most frequently deflection or delayed eruption. Here, the reported study details the surgical management of a mandibular compound odontoma in a patient who presented with a complaint of numbness in his lower jaw.

## Introduction

In the category of odontogenic abnormal growths, odontomas are the most common non-cancerous structures of mixed origin, according to the World Health Organization [1]. Odontomas are usually diagnosed in the first 20 years of life and are gender-insensitive [2]. Odontomas are a little more common in men than in women. The maxilla has a higher prevalence of odontomas (67%) with a clear preference for the anterior maxillary area (61%) [3]. Although the exact cause of these lesions is still unknown, some pathological conditions have been linked to them, including inflammation, immature ameloblasts, hereditary anomalies (Gardner’s syndrome, Hermanns syndrome), odontoblastic hyperactivity, and changes in the genetic component that regulates dental development [1]. Odontomas can also result from local injury to the developing tooth germ [4].

Odontomas are frequently found on regular dental radiographs and are usually asymptomatic [5]. Even though odontomas are rarely bigger than teeth, they might cause expansion of the cortical bone [6]. Retaining primary teeth, failing permanent teeth to erupt, discomfort, expansion of the outer layer of bone, and tooth displacement are all indications of odontomas [7]. There may be other symptoms, such as headaches in the front of the head, swelling in the affected areas, and numbness in the lower lip [8]. Pain is rare and typically results from a secondary infection brought on by oral bacteria getting into the area between the odontoma and the bone [9].

Compound and complex odontomas are the two different categories of odontomas. A deformity known as a compound odontoma is one in which every tooth tissue is grouped in a well-organized manner that is specific to little teeth. On the other hand, an irregular mass grouped in an irregular pattern is referred to as a "complex odontoma" [10]. It is significant to remember that odontomas are often non-aggressive and slow-growing. Nonetheless, in order to prevent problems with tooth eruption, prompt detection and enucleation of these hamartomas are advised [11].

## Case presentation

The 24-year-old male patient presented at King Saud University Dental Hospital with a chief complaint of occasional numbness in his lower right jaw. His medical history revealed no remarkable findings. After visiting a private clinic, a panoramic radiograph identified a retained deciduous lower right canine, and the patient was advised to schedule an appointment with a surgeon.

Intraoral examination indicated a swelling over the buccal gingiva, extending from the mesial margin of the canine to the labial frenum. The patient reported numbness, describing it as a "tingling sensation" that began over two months.

**Figure 1:**
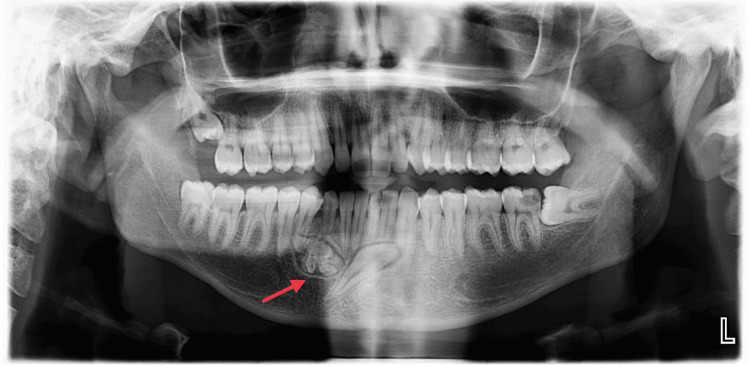
Panoramic radiograph showing the presence of an odontoma in the anterior mandibular area with impacted canine.

Radiographic assessments, including panoramic and cone-beam computer tomography, revealed an impacted lower right permanent canine in a mesioangular position. A large, well-corticated lesion with a radiolucent border encapsulating tooth-like nodular masses was highly suggestive of a compound odontoma. The lesion was located below the root of the retained primary lower right canine and the lower first premolar (Figure 1).

**Figure 2:**
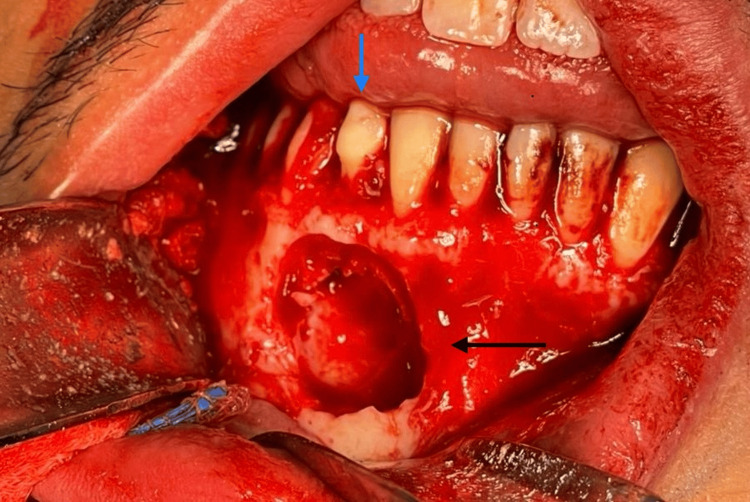
Intraoral view following removal of the odontoma (black arrow). Note that the primary canine was kept in place (blue arrow).

Under general anesthesia via nasal intubation and local anesthesia with epinephrine at the surgical site, a sulcular incision was made in the buccal region from teeth #48 to #36. The flap was reflected by an oral surgeon, and the right-side mental nerve was identified and protected. Tooth #48 was extracted, and access to the odontoma was gained by removing the overlying bone with a bur under copious saline irrigation. The odontoma was successfully removed, along with the extraction of the lower right permanent canine while preserving the primary right canine (Figure 2). Simultaneously, the lower third molars were also extracted, eliminating the need for a separate surgery. Bleeding in the odontoma area was controlled with bone wax and sutured with Vycrill 4-0. The patient received a prescription for oral antibiotics and analgesics post-surgery.

**Figure 3:**
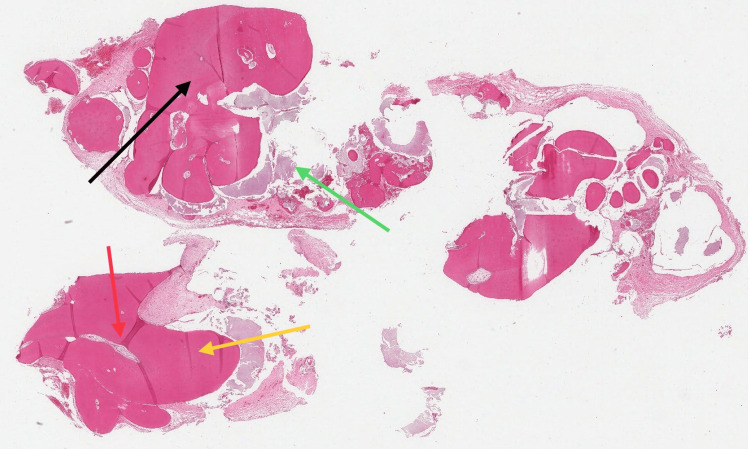
Histologic section of odontoma showing: reduced enamel epithelium (black arrow), enamel matrix (green arrow), pulp-like structure (red arrow), dentin-like structure (yellow arrow).

Histologic examination confirmed the compound odontoma diagnosis, revealing well-delineated, roughly spherical masses of haphazard hard dental tissue partially surrounded by fibrous connective tissue. The dental tissue comprised tubular dentin-enclosing zones of enamel matrix with a fish-scale-like appearance and/or fibrous tissue rimmed by odontoblasts. Additionally, there was reduced enamel epithelium forming a cyst-like pattern containing ring-like basophilic (psammomatoid) calcifications, small islands of eosinophilic-staining epithelial ghost cells, and odontogenic islands with clear cells (Figure 3).

**Figure 4:**
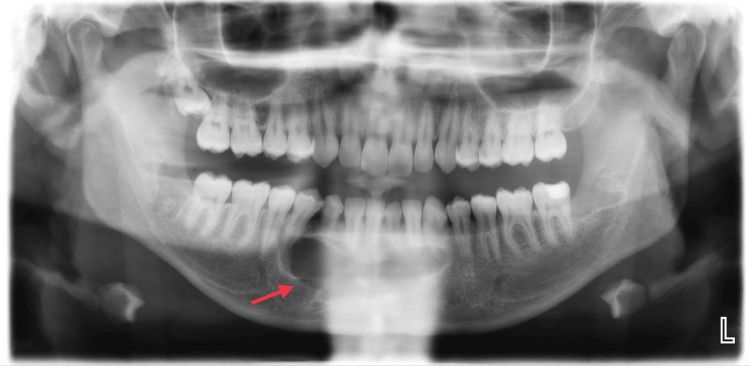
Panoramic radiograph after surgically removed odontoma and permanent canine.

**Figure 5:**
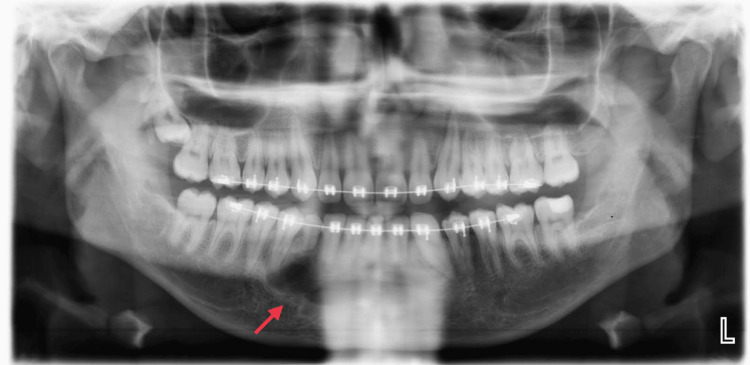
Panoramic radiograph after six months followed up.

The patient's recovery proceeded smoothly, with the surgical site healing excellently and numbness improving. Upon completion of the six-month follow-up, there were no radiological or clinical complications. Furthermore, no issues or indications of a recurrence were noted during the ensuing follow-up visits (Figures 4-5).

## Discussion

Most odontomas are commonly diagnosed during routine radiographic examinations [12]. Based on the radiographic findings of panoramic and cone beam computer tomography, the case recorded in this report was originally diagnosed as a compound odontoma. The lesion's histopathological analysis later supported this diagnosis. Odontomas are mostly asymptomatic; however, lower jaw numbness is one symptom that may develop if it happens in the mandible [8].

The radiographic findings of odontomas are determined by their stage of development and degree of mineralization. The first stage is characterized by radiolucency due to the lack of calcification. Partial calcification appears in the intermediate stage, while in the third stage, the lesion usually presents as radiopaque masses surrounded by radiolucent areas corresponding to the connective tissue histologically [12].

Most cases of odontoma are detected when the permanent teeth fail to erupt or when a primary tooth is retained, and the majority of these cases are treated with a combination of orthodontic and surgical procedures [13]. In the present circumstance, the impacted canine was surgically removed to avoid future problems, and the surgical procedure was planned based on the position of the impacted canine, as exposure and subsequent orthodontic treatment were not recommended [14].

Although odontoma has little potential for growth, it should be removed because it contains different tooth formations that can interfere with the eruption of permanent teeth, cause significant bone loss, and predispose to cystic change [12]. Despite the uncommon nature of this lesion and the fact that the majority of cases are surgically removed and healed without complications, there needs to be careful monitoring, as there have been reports of associations with adenomatoid tumors, ameloblastomas, and carcinoma [15].

Research has indicated that compound odontomas are more common in the anterior maxilla. An odontoma was discovered in the anterior mandible in the current case, which is an unusual location for it to exist. The area around the canines and incisors had been linked to an upsurge in the number of odontomas in earlier publications; in this instance, there was a lower frequency [16,17].

Clinicians should be aware of conditions such as orofacial and systemic malignancies, as well as various inflammatory disorders, which can contribute to numbness in the lower jaw. Temporally associated dental causes are another issue to consider. A thorough diagnostic assessment is essential in such cases [18].

## Conclusions

A routine panoramic radiography and clinical examinations are recommended for the early detection of odontomas, particularly when there are retained primary teeth or delayed eruption of permanent teeth. Lower jaw numbness is especially important to consider, as patients and medical professionals sometimes minimize or disregard it. It is advisable to diagnose odontomas and proceed with surgical enucleation followed by curettage. It is worth noting that odontomas generally have a good prognosis with rare relapses.
